# A New Peptide Ligand for Targeting Human Carbonic Anhydrase IX, Identified through the Phage Display Technology

**DOI:** 10.1371/journal.pone.0015962

**Published:** 2010-12-31

**Authors:** Vasileios Askoxylakis, Regine Garcia-Boy, Shoaib Rana, Susanne Krämer, Ulrike Hebling, Walter Mier, Annette Altmann, Annette Markert, Jürgen Debus, Uwe Haberkorn

**Affiliations:** 1 Department of Radiooncology and Radiation Therapy, University of Heidelberg, Heidelberg, Germany; 2 Department of Nuclear Medicine, University of Heidelberg, Heidelberg, Germany; 3 Clinical Cooperation Unit Nuclear Medicine, German Cancer Research Center, Heidelberg, Germany; University of South Florida College of Medicine, United States of America

## Abstract

Carbonic anhydrase IX (CAIX) is a transmembrane enzyme found to be overexpressed in various tumors and associated with tumor hypoxia. Ligands binding this target may be used to visualize hypoxia, tumor manifestation or treat tumors by endoradiotherapy.

**Methods:**

Phage display was performed with a 12 amino acid phage display library by panning against a recombinant extracellular domain of human carbonic anhydrase IX. The identified peptide CaIX-P1 was chemically synthesized and tested *in vitro* on various cell lines and *in vivo* in Balb/c nu/nu mice carrying subcutaneously transplanted tumors. Binding, kinetic and competition studies were performed on the CAIX positive human renal cell carcinoma cell line SKRC 52, the CAIX negative human renal cell carcinoma cell line CaKi 2, the human colorectal carcinoma cell line HCT 116 and on human umbilical vein endothelial cells (HUVEC). Organ distribution studies were carried out in mice, carrying SKRC 52 tumors. RNA expression of CAIX in HCT 116 and HUVEC cells was investigated by quantitative real time PCR.

**Results:**

*In vitro* binding experiments of ^125^I-labeled-CaIX-P1 revealed an increased uptake of the radioligand in the CAIX positive renal cell carcinoma cell line SKRC 52. Binding of the radioligand in the colorectal carcinoma cell line HCT 116 increased with increasing cell density and correlated with the mRNA expression of CAIX. Radioligand uptake was inhibited up to 90% by the unlabeled CaIX-P1 peptide, but not by the negative control peptide octreotide at the same concentration. No binding was demonstrated in CAIX negative CaKi 2 and HUVEC cells. Organ distribution studies revealed a higher accumulation in SKRC 52 tumors than in heart, spleen, liver, muscle, intestinum and brain, but a lower uptake compared to blood and kidney.

**Conclusions:**

These data indicate that CaIX-P1 is a promising candidate for the development of new ligands targeting human carbonic anhydrase IX.

## Introduction

The outcome of cancer treatment can be influenced by the microenvironment within a solid tumor. One of the factors that influence tumor progression is hypoxia. Tumor hypoxia is associated with a less favourable phenotype, characterized by high invasiveness, increased potential for metastasis and poor prognosis, resulting in reduced overall survival [1], [2]. Subphysiologic levels of oxygen in the tumor lead to an up to 3-fold increase of resistance against antineoplastic therapy [3]. Furthermore, tumor hypoxia influences the migration activity of endothelial cells, resulting in an amplified signalling for angiogenesis [4].

Low oxygen tension results in the activation of a series of transcriptional regulators including hypoxia inducible factor 1 (HIF-1) [5]. HIF-1 has a central role as oxygen threshold in mammalian cells. Under hypoxic conditions, HIF-1 binds to hypoxia response elements (HRE) of its target genes and induces their expression [6]. One of the inducible targets of HIF-1 transcriptional activity is carbonic anhydrase IX (CAIX) [7]. CAIX is a member of a family of zinc metalloenzymes, which catalyse the hydration of carbon dioxide into carbonic acid. It is a membrane associated glycoprotein, consisting of an extracellular catalytic domain extended with a proteoglycan-like region, a transmembrane anchor and a short C-terminal cytoplasmic tail [8]. The protein is found to be overexpressed in various human tumors, such as carcinomas of the colon, kidney and lung [9], [10], [11], and various clinical studies have demonstrated a correlation between CAIX expression and disease prognosis [12], [13].

The leading role of tumor hypoxia in increased therapy resistance reveals the necessity for the development of hypoxia imaging assays. Such assays would allow a better characterization of tumor heterogeneity in respect of oxygenation, which is important for targeted therapies, and the development of strategies for predicting treatment outcome. In this respect, radiolabeled nitroimidazole compounds find wide clinical application [14]. These compounds are reduced by intracellular reductases into reactive metabolites, which subsequently bind to thiol groups of intracellular proteins, resulting in accumulation within hypoxic cells [15].

Still, there is increasing interest in the development of molecular imaging strategies based on ligands that bind selectively to target proteins overexpressed at hypoxic sites. The fact that CAIX represents an endogenous marker for cellular hypoxia with predictive potential and that it is easily accessible through its extracellular domain make carbonic anhydrase IX to an attractive molecule for targeting approaches. A further interesting feature is its strong overexpression in renal cell carcinoma. Monoclonal antibodies with high affinity to human carbonic anhydrase IX have already been generated and tested for diagnosis as well as for treatment [16], [17], [18].

Peptides are an attractive alternative to antibodies. They possess favourable pharmacokinetic properties through their small size, such as rapid clearance from blood, while lacking the immunogenic potential of antibodies. Furthermore, peptides are easy and cheap to synthesize. Therefore, there is increasing interest in the development of new peptide ligands with specific targeting abilities.

A very promising tool for the identification of new specific binders is the phage display technology. The method has found wide application for the identification of new receptors and natural ligands, mapping and mimicking epitopes or isolating specific antigens that bind to bioactive compounds [19]. Phage display was also successfully applied for the selection of novel peptides that target organs, tumors or cell types [20], [21].

In this study we applied the phage display technology for the identification of a new peptide ligand binding specifically to human carbonic anhydrase IX. Panning was performed using the recombinant extracellular domain of CAIX as target structure. The identified peptide CaIX-P1 was synthesized and its binding properties were evaluated *in vitro* on various cell lines. Furthermore, *in vivo* organ distribution studies in tumor bearing mice were performed and the stability of the peptide in human serum was investigated.

## Methods

### Cell lines

All cell lines were cultivated at 37°C in a 5% CO_2_ incubator. The human renal cell carcinoma cell line SKRC 52 was obtained by O. Boerman (Univ. of Nijmegen, The Netherlands). SKRC 52 and CaKi 2 cells, as well as the human colorectal carcinoma cell line HCT 116 were cultured in RPMI 1640 with GlutaMAX (Invitrogen) containing 10% (v/v) fetal calf serum (Invitrogen). Primary isolated human umbilical vein endothelial cells (HUVEC: Promocell, Heidelberg, Germany) were cultured in serum reduced (5% fetal calf serum [FCS]) modified Promocell medium (MPM), supplemented with 2 ng/mL VEGF and 4 ng/mL basic fibroblast growth factor (bFGF).

### Recombinant isolation of the extracellular domain of carbonic anhydrase IX

For recombinant isolation of the extracellular domain of human carbonic anhydrase IX (CAIX) the Flp-In system (Invitrogen life technologies) was used. The gene encoding for human carbonic anhydrase IX inserted into a pCMV6-XL5 vector was obtained from Origene, Rockville. The primers for PCR amplification of the sequence encoding for the extracellular domain of CAIX were forward: 5′-AAC TTA AGC TTG GGG CCG CCA CCA TGG CTC CCC TGT GCC CCA-3′ and reverse: 5′-GGC TCC GGA TCC ATG TCC CTG CCC TCG ATG TCA CCA GCA GCC AGG CAG-3′. After PCR amplification, the sequence encoding for the extracellular domain of CAIX was inserted into the HindIII and BspEI sites of pSEC-EGP-2-Fcγ vector (Affimed, Heidelberg). The fragment CAIX-Fc was cut by HindIII and XhoI and inserted into pcDNAEpcam vector (Affimed, Heidelberg). This vector was cotransfected with the Flp recombinase expression vector pOG44 into the Flp-In™-293 human embryonic kidney host cell line, as described in the Flp-In protocol. Thereafter, a selection for hygromycin resistant cells was performed. The expressed protein was isolated and purified from the incubation medium through a HiTrap™MabSelect SuRe™ column. Qualitative control was performed by ELISA and western blot analysis (data not shown).

### Selection of peptides binding carbonic anhydrase IX

A linear 12-amino acid peptide library (Ph.D.12; New England Biolabs) was used for biopanning. Panning was performed on immobilized recombinant extracellular domain of human carbonic anhydrase IX. Immobilized recombinant extracellular domain of the epidermal growth factor receptor (EGFR) was used for negative selection. For immobilization protein was incubated in Maxisorp plates (50 nM) for 24 h at 4°C. Each selection round was conducted as follows: 10^11^ plaque-forming units were added on immobilized negative target (EGFR) in 96well plates. After 1 h incubation at room temperature medium was transferred in 96-wells containing the immobilized positive target (CAIX). Incubation was carried out for 1 h at room temperature. Subsequently, medium was removed and the target was washed 10 times with 100 µl Tris Buffered Saline with Tween (TBST) pH 7.4. Elution of the bound phages was performed through incubation for 10 min with 10 µl 0.2 M glycine/HCl buffer pH 2.2, containing 1 mg/mL bovine serum albumin (BSA) at room temperature. After neutralization with 15 µl Tris/HCl buffer pH 9.1, centrifugation was performed for 5 min at 1000 rpm. Supernatant was collected and 10 µl were used for phage titration on isopropyl β-D-1-thiogalactopyranoside/X-Gal (IPTG/X-Gal) (Fermentas) lysogeny broth agar plates. The remaining supernatant was used for amplification in 20 mL of ER2537 bacteria according to the manufacturer's protocol. After 7 selection rounds, clones were picked and phage single-stranded isolation was performed (QIAprep Spin M13 Kit; Qiagen). DNA sequencing was carried out and the displayed peptide was identified through analysis with the HUSAR map (HUSAR Biocomputing Service at the German Cancer Research Center, Heidelberg, Germany).

### Peptides

The peptide CaIX-P1 (YNTNHVPLSPKY) and its derivative CaIX-P1-2-12 (NTNHVPLSPKY) were obtained by solid phase peptide synthesis using Fmoc coupling protocols. CaIX-P1 and CaIX-P1-2-12 were synthesized on an ABI 433 A peptide synthesis reactor (Applied Biosystems). The peptides were purified by high performance liquid chromatography (HPLC) on a Chromolith Semi Prep Column RPe18, 10×100 mm (Merck, Darmstadt, Germany), with a linear gradient of water and acetonitrile containing 0.1% trifluoroacetic acid and subsequent lyophilization. The mass of the products was determined by mass spectrometry analysis on a matrix-assisted laser desorption ionization time-of-flight mass spectrometer (MALDI-3; Kratos instruments). Labeling with ^125^I and ^131^I was performed using the chloramine-T method [22]. The iodinated product was purified and analyzed on a Chromolith Performance RP-18e 100×4.6 mm column (Merck) using a linear gradient of water and acetonitrile containing 0.1% trifluoroacetic acetic acid. The iodination product of CaIX-P1 was a mixture of iodinated species that could not be separated from each other due to their similar retention times in the HPLC chromatograms. All experiments were conducted under the same radiolabeling conditions.

### Binding experiments on immobilized protein

Binding of ^125^I-labeled CaIX-P1 was performed on immobilized recombinant extracellular domain of human CAIX and of EGFR. For immobilization the target proteins CAIX and EGFR were incubated at a concentration of 50 nM in 24-well plates for 24 h. Thereafter, the plates were washed three times with 500 µl PBS pH 7.4. Incubation with ^125^I-CaIX-P1 was performed in 500 µl PBS pH 7.4 for 30 min. After incubation the plates were washed three times with 500 µl ice cold PBS pH 7.4. The target proteins were degraded with 500 µl NaOH 0.3 mol/L and the radioactivity was counted with a γ-counter. Bound radioactivity was calculated as percentage applied dose. In order to evaluate the specificity of the radioligand binding, competition experiments with the unlabeled CaIX-P1 peptide at a concentration of 10^−4^ M were carried out.

### *In vitro* binding experiments

For binding experiments 3×10^5^ CAIX positive SKRC 52 cells were seeded into 6-well plates and cultivated in 3 mL of incubation medium at 37°C for 24 h. After cell blocking with RPMI 1640 (without FCS) containing 1% BSA, the medium was replaced with 1 mL of fresh medium (without FCS) containing 0.5–1.5×10^6^ cpm of ^125^I-labeled peptide and incubation was performed for time periods varying from 10 min to 6 h at 37°C. To determine specific versus nonspecific binding, the cells were incubated with the unlabeled CaIX-P1 peptide at concentrations varying from 10^−4^ to 10^−10^ mol/L. Octreotide was used as negative control competitor. After incubation the medium was removed and the cells were washed three times with 1 mL ice cold PBS in order to remove the unbound radiolabeled peptide. Subsequently, the cells were lysed with 0.5 mL NaOH 0.3 mol/L and the radioactivity was measured with a γ-counter. Bound radioactivity was calculated as percentage applied dose per 10^6^ cells. Binding experiments were also performed on HCT 116 cells and on human umbilical vein endothelial cells (HUVEC) at various cell densities. CAIX negative CaKi-2 cells were used as negative control.

Binding kinetic of the ^125^I-labeled CaIX-P1-2-12 was performed on SKRC 52 cells for incubation periods varying from 10 min to 2 h at 37°C.

### Internalization studies

Subconfluent cell cultures of SKRC 52 cells were incubated with ^125^I-CaIX-P1 for 10 min, 60 min, 120 min and 240 min at 37°C and 4°C. Cellular uptake was stopped by removing the medium and washing three times with 1 mL PBS. Subsequently, cells were incubated with 1 mL of glycine-HCl 50 mmol/L in PBS (pH 2.8) for 10 min at room temperature, in order to remove the surface bound activity. The cells were then washed with 3 mL of ice-cold PBS and lysed with 0.5 mL of NaOH 0.3 mol/L. The surface and the internalized radioactivity were measured with a γ-counter and calculated as % applied dose per 10^6^ cells.

### Stability studies

The stability of CaIX-P1 was investigated in human serum. The peptide was incubated at 37°C in human serum at a concentration of 10^−4^ mol/L. At time points varying from 5 min to 2 h aliquots were taken, mixed with equal volume acetonitrile to precipitate serum proteins and centrifuged for 5 min at 13,000 rpm. The supernatant was analyzed with HPLC on a Chromolith Performance RP-18e 100×4.6 mm column (Merck) using a linear gradient of water and acetonitrile containing 0.1% trifluoroacetic acid. Samples of CaIX-P1 and its fragments in human serum were isolated and analyzed by MALDI-TOF mass spectrometry.

### *In vivo* experiments

Organ distribution studies were performed in 9-week-old female Balb/c nu/nu mice, carrying subcutaneously transplanted SKRC 52 tumors. Animals were obtained from Charles River WIGA and housed in VentiRacks (BioZone Global). A cell suspension of 4×10^6^ cells in OPTI-MEM (Gibco, Invitrogen Life Technologies) was injected subcutaneously into the mouse flank and the tumors were grown to a size of 1.0 cm^3^. ^131^I-labeled CaIX-P1 was injected into the tail vein of the animals (approximately 1 MBq) and at 15 min, 60 min and 240 min after injection the animals were sacrificed. Tumor, blood and selected tissues (heart, spleen, liver, kidney, muscle, intestinum and brain) were removed, drained of blood, weighed and the radioactivity was measured in a γ-counter (LB 951G; Berthold Technologies) Also 3 aliquots of the tracer solution used for injection were measured. The organ uptake was calculated as percentage injected dose per gram tissue (% ID/g). In order to investigate the *in vivo* specificity, blocking experiments were performed. For *in vivo* blocking, the radioligand was co-injected with 100 µL unlabeled CaIX-P1 peptide at a concentration of 10^−3^ M and organ distribution was carried out 15 min and 60 min after intravenous application.

For determination of the chemical form of the circulating radioactivity in the blood of the animals, samples of blood were taken at 1 h post injection, centrifuged and serum analysis was performed by HPLC.

All animal experiments were carried out in conformity with the German law for protection of animals and are in compliance with European laws. Study approval was received by the Regierungspräsidium Karlsruhe, Abteilung 3, Baden-Württemberg, Germany, File reference: 35-9185.81/G-132/04.

### Real time quantitative PCR

Total cellular RNA was isolated from confluent HCT 116 and HUVEC cells in 10 cm cell culture dishes using the Trizol method (TRIzol Reagent, Invitrogen). Cellular RNA was also isolated from HCT 116 cells in 6-wells at various cell densities. RNA extraction was carried out with a standard phenol-chloroform protocol. RNA concentration was measured with a NanoDrop spectrophotometer (ND-1000 PeqLab Biotechnologie GmbH, Germany). 500 ng was transcribed into DNA using M-MLV reverse transcriptase, 50 pmol random hexamer and 100 pmol of oligo(dT) primers (Promega, Madison, WI, USA). The LightCycler FastStart DNA Master Hybridization Probes kit was used for quantification of relative mRNA transcript levels on a Light Cycler (Roche Applied Sciences), applying the TaqMan methodology. Normalization was performed using β2-microglobulin as house keeping gene. Primers were obtained from Applied Biosystems (Foster City, CA, USA).

### Statistics

Statistical comparisons between groups were performed by the Student's t test using the SIGMASTAT program (Jandel Scientific, Erkrath, Germany). A p value of 0.05 or less was considered statistically significant.

## Results

### Selection of peptides binding the extracellular domain of carbonic anhydrase IX

In order to identify human carbonic anhydrase IX specific binding peptides, phages expressing 12mer peptides on their surface were applied to *ex vivo* selection rounds on immobilized recombinant extracellular domain of CAIX. Recombinant extracellular domain of the epidermal growth factor receptor (EGFR) was used for negative control selection. After 7 selection rounds, single-stranded DNA from isolated bound phages was sequenced. All 20 clones sequenced, displayed the same peptide sequence: YNTNHVPLSPKY (CaIX-P1).

### Binding experiments on immobilized protein

**Figure 1:**
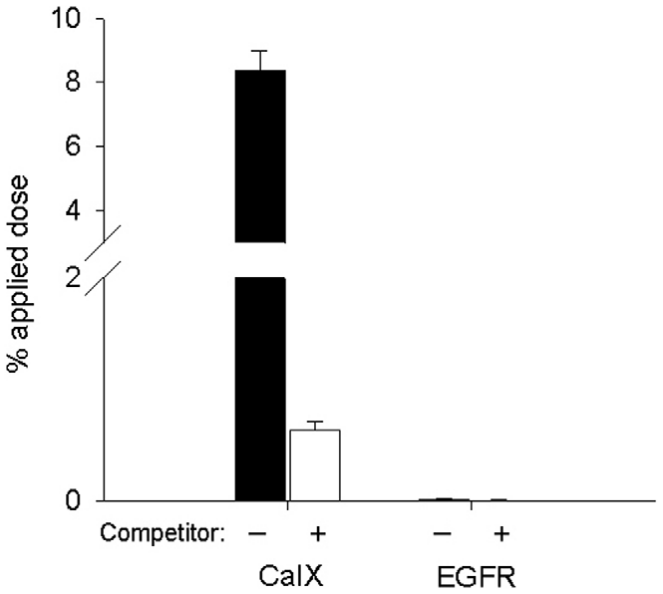
Binding and competition of ^125^I-labeled CaIX-P1 on the recombinant extracellular domain of CAIX and EGFR. Non specific binding was determined in the presence of 10^−4^ M unlabeled CaIX-P1. Mean values and standard deviation (n = 3).

Binding of CaIX-P1 was investigated on immobilized recombinant target protein. As negative control immobilized recombinant EGFR protein was used. Binding of the radioligand was 8.5% on the extracellular domain of CAIX. Co-incubation of the radioligand with the unlabeled CaIX-P1 peptide at a concentration of 10^−4^ M led to a binding inhibition of 93% (p<0.05). Experiments on the negative control target (EGFR) revealed a reduced binding to the background level (p<0.05) (Figure 1).

### *In vitro* binding and competition experiments

**Figure 2:**
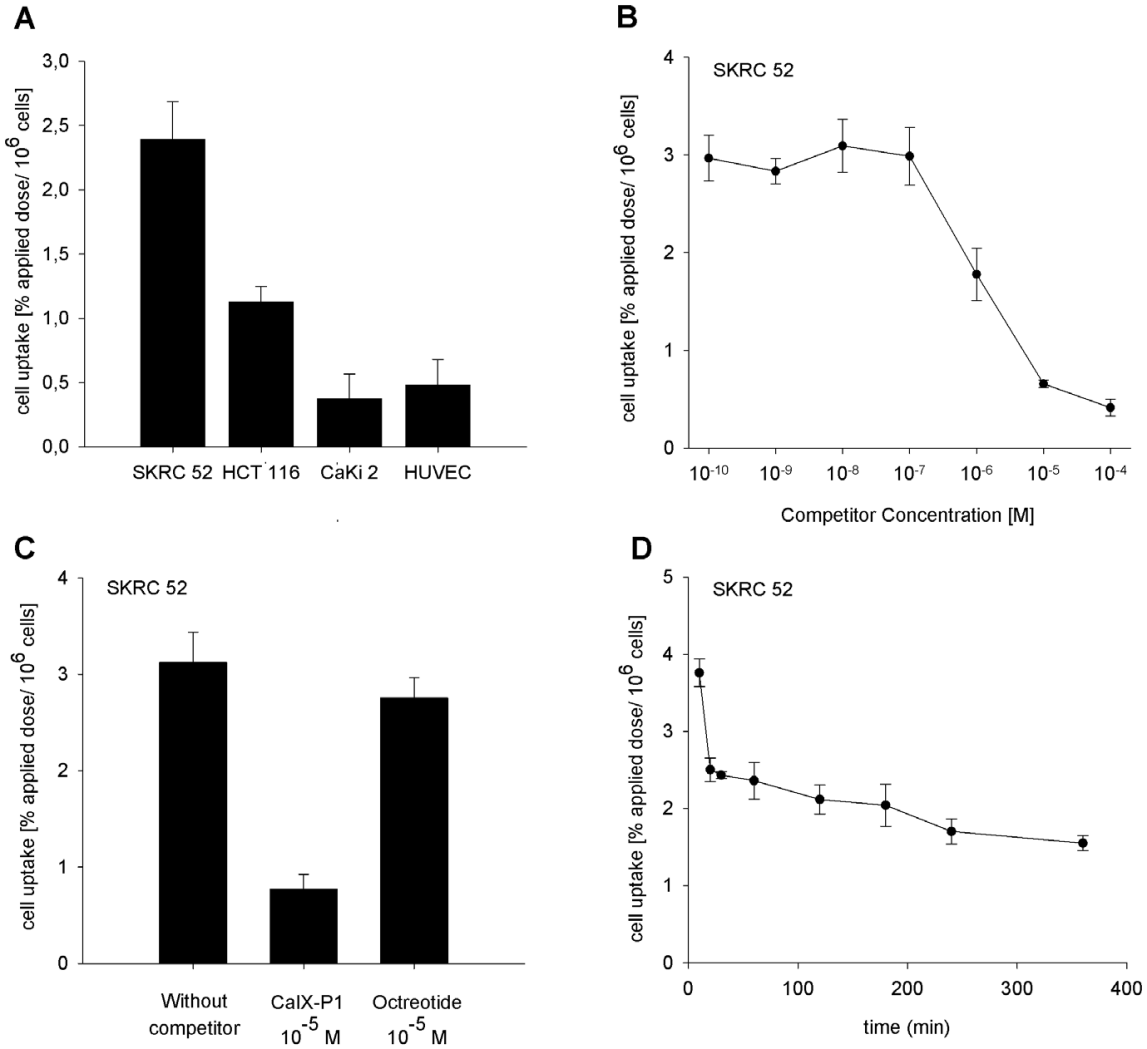
*In vitro* characterization of ^125^I-labeled CaIX-P1. A: Binding of ^125^I-labeled CaIX-P1 in the CAIX positive human renal cell carcinoma cell line SKRC 52, the human colorectal carcinoma cell line HCT 116, the CAIX negative human renal cell carcinoma cell line CaKi 2 and on human umbilical vein endothelial cells (HUVEC). B: Displacement of bound ^125^I-CaIX-P1 by the unlabeled CaIX-P1 peptide at various concentrations in SKRC 52 cells. C: Specific binding of ^125^I-CaIX-P1 in SKRC 52 cells. Non specific binding was determined in the presence of 10^−5^ M unlabeled CaIX-P1. Octreotide was used at the same concentration (10^−5^ M) as negative control competitor. D: *In vitro* cell accumulation of ^125^I-CaIX-P1 in SKRC 52 cells as a function of time. Incubation was performed for time periods from 10 min to 360 min. Mean values and standard deviation (n = 3).

*In vitro* binding of CaIX-P1 was investigated on various cell lines. Cells were incubated with the radioligand in serum free medium in order to avoid peptide degradation. The *in vitro* binding experiments demonstrated the highest uptake for the CAIX positive renal cell carcinoma cell line SKRC 52. In particular, the binding capacity in SKRC 52 cells was about 2.5% applied dose per 10^6^ cells after 60 min incubation with the radioligand. Binding of ^125^I-CaIX-P1 in the colorectal carcinoma cell line HCT 116 was 1.0 to 1.5%. Performing binding experiments in the CAIX negative human renal cell carcinoma cell line CaKi 2 and in human umbilical vein endothelial cells (HUVEC), the binding capacity was found to be reduced to the background level with 0.4% of the applied dose per 10^6^ cells on CaKi 2 and HUVEC cells (Figure 2A). The difference in the binding capacity between the positive tumor cell lines SKRC 52 and HCT 116 and the negative control cell lines CaKi 2 and HUVEC was found to be highly significant with p<0.01.

Co-incubation of ^125^I-labeled CaIX-P1 with the unlabeled peptide in SKRC 52 cells resulted in a concentration dependent inhibition of the radioligand binding with a maximal inhibition of 90% at 10^-4^ mol/L (p<0.01) and an IC50 value of 1.75±0.49 µM (Figure 2B). Using octreotide as negative control competitor at the same concentration the uptake of ^125^I-CaIX-P1 on SKRC 52 cells was not influenced (Figure 2C).

### Kinetic studies

Kinetic studies of ^125^I-CaIX-P1 in SKRC 52 cells, with incubation periods varying from 10 min to 6 h, revealed a time dependent decrease of the radioligand uptake. Particularly, maximal uptake of 3.8% was reached after an incubation period of 10 min. Thereafter a time-dependent decrease was noticed with the bound activity reaching a value of about 1.6% after 6 h of incubation (Figure 2D).

### Internalization studies

**Figure 3:**
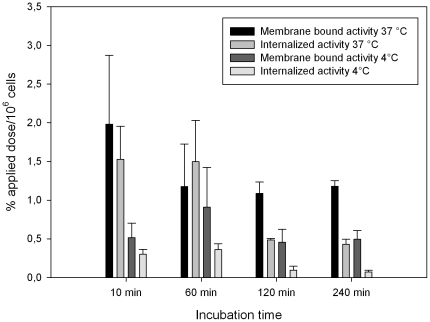
Binding and internalization of ^125^I-CaIX-P1 in SKRC 52 cells. Cells were incubated with the radioligand for 10 min, 60 min, 120 min and 240 min at 37°C or at 4°C. Mean values and standard deviation (n = 3).

To distinguish between surface bound and internalized peptide, *in vitro* internalization was investigated in SKRC 52 cells. For those studies the surface bound peptide was removed by including an acidic wash step in the washing procedure. After 10 min incubation with ^125^I-CaIX-P1 at 37°C, the internalized radioactivity was measured as about 45% of the total bound activity, while after 60 min incubation about 60% of the total uptake was found to be internalized into the SKRC 52 cells. For longer incubation periods of 120 and 240 min internalized activity was reduced to about 30% of the total uptake. Internalization experiments were also performed at 4°C demonstrating a reduction of both total and internalized radioactivity (p<0.05). The measured internalized activity at 4°C was reduced to the background level of <0.3% applied dose per 10^6^ cells (Figure 3).

### Quantitative real time PCR and ^125^I-CaIX-P1 binding in HCT 116 and HUVEC cells

**Figure 4:**
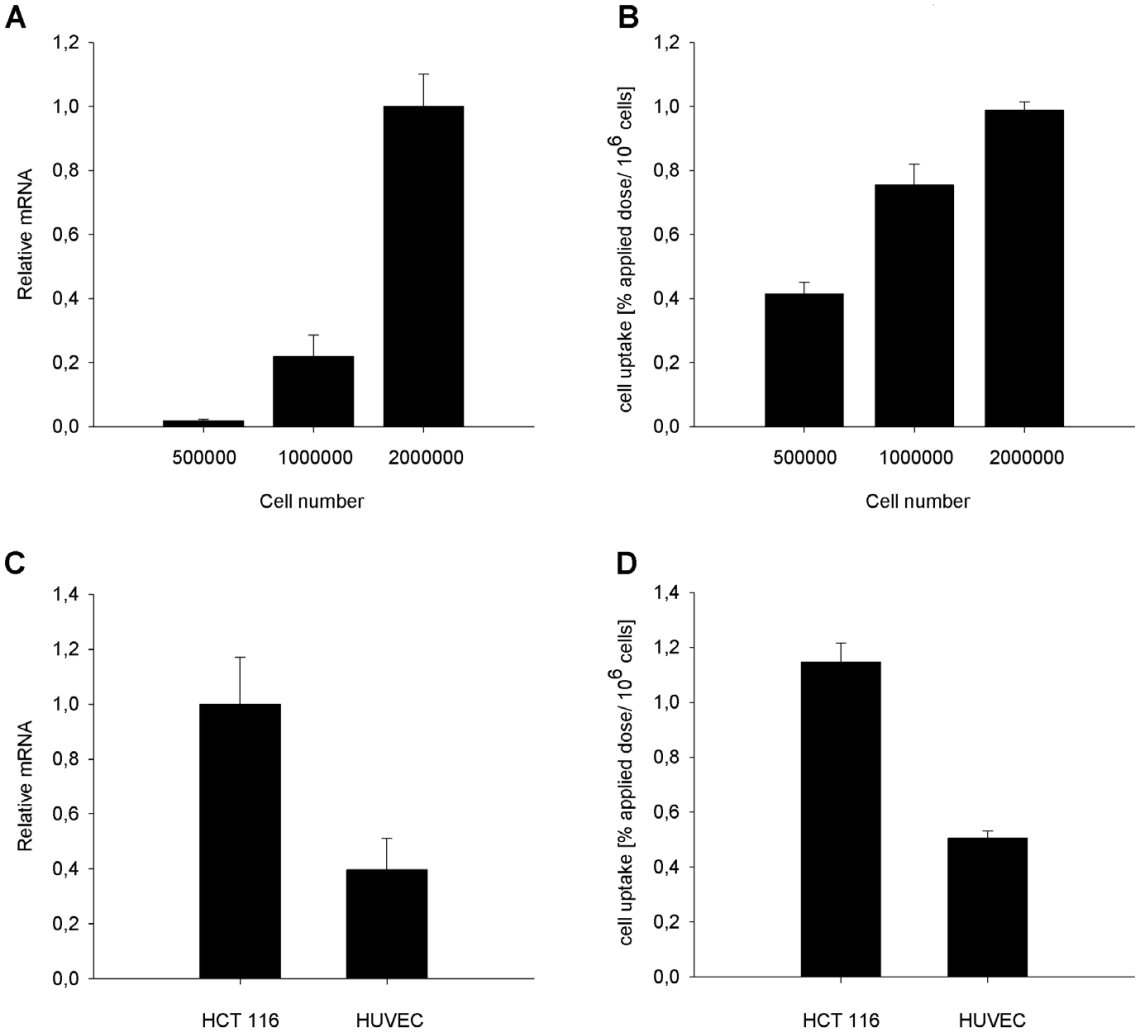
Quantitative RT-PCR analysis of CAIX mRNA in HCT 116 and HUVEC cells. A: CAIX mRNA levels in HCT 116 cells as function of the cell density. B: Binding of ^125^I-CaIX-P1 in HCT 116 cells as function of the cell density. C: CAIX mRNA levels in HCT 116 and HUVEC cells at the same cell density. D: Binding of ^125^I-CaIX-P1 in HCT 116 and HUVEC cells at the same density. Mean values and standard deviation (n = 3).

Quantitative real time PCR analysis showed an upregulation of CAIX mRNA in the human colorectal carcinoma cell line HCT 116 with increasing cell density (Figure 4A). The mRNA content correlated with the CaIX-P1 uptake in HCT 116 cells. In particular, radioligand binding was found to increase with increasing cell number in the 6-well plate (p<0.05) (Figure 4B).

qRT-PCR analysis demonstrated higher CAIX mRNA levels for HCT 116 cells compared to HUVEC cells at same density (Figure 4C), which also correlated to the CaIX-P1 binding in the two cell lines (p<0.05) (Figure 4D).

### Stability in human serum

**Figure 5:**
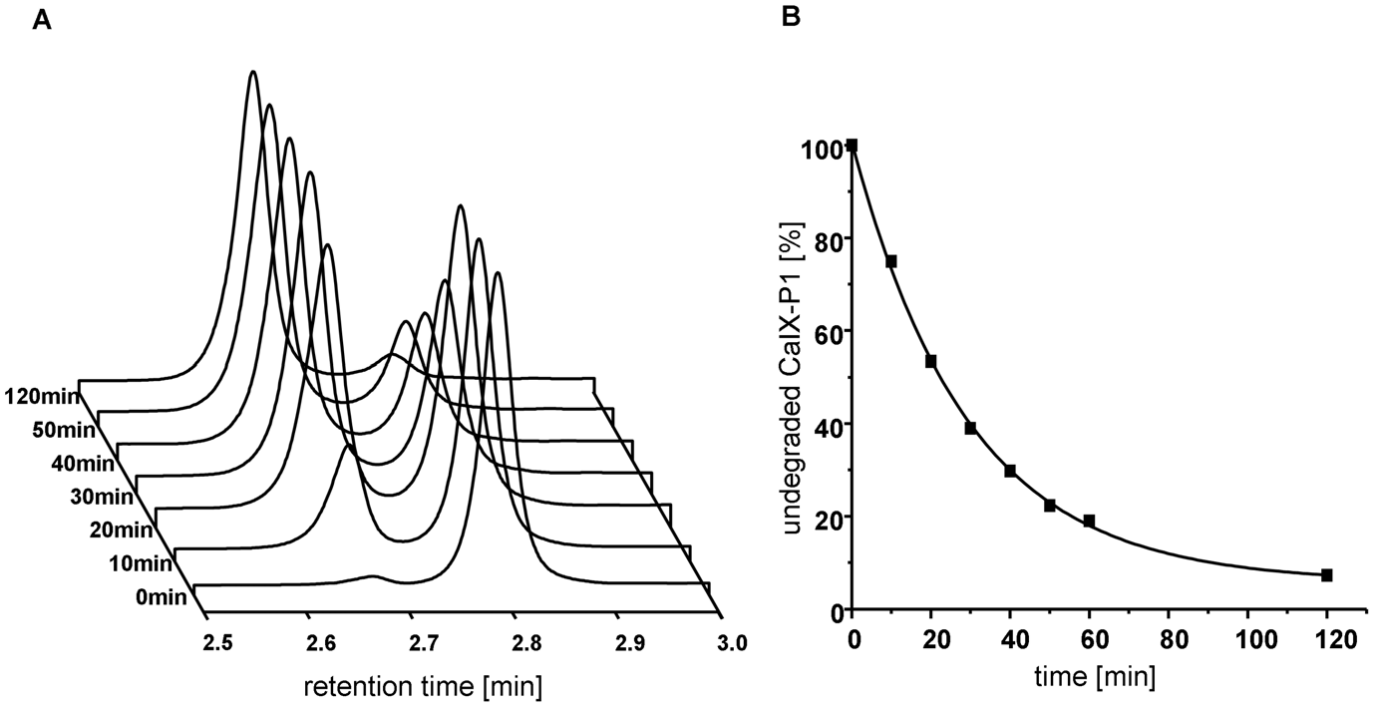
Serum stability analysis of CaIX-P1. A: HPLC analysis of aliquots collected at time points from 0 min to 120 min. The right peak represents the peptide CaIX-P1 and the left peak represents the first product of the peptide degradation in serum. B: Serum stability in human serum at 37°C.

The *in vitro* stability of CaIX-P1 in human serum was investigated through incubation of the peptide at a concentration of 10^−4^ mol/L in human serum and HPLC of samples taken at different time points. The experiments revealed a degradation of the peptide through serum proteases over time (Figure 5A). The first degradation product of CaIX-P1 identified by HPLC was isolated and mass spectrometry was performed in order to identify the site of cleavage. The main product of serum degradation appeared after 10 min incubation of the peptide in serum and had a mass of 1270 g/mol. This mass corresponds to an 11 amino acid sequence, lacking the N-terminal tyrosine (CaIX-P1-2-12). Analysis of the samples revealed a half-life of CaIX-P1 of about 25 min (Figure 5B).

### In vitro kinetics of ^125^I-labeled CaIX-P1-2-12

Binding of the first metabolic product of CaIX-P1 in human serum (CaIX-P1-2-12) was investigated in SKRC 52 cells. Kinetics of ^125^I-CaIX-P1-2-12 in the CAIX positive cell line revealed a time dependent increase of the radiolgand uptake for an incubation period of 30 min. Thereafter, only a slight decrease of the radioligand uptake was noticed for an incubation period of up to 2 h (Figure S1).

### Organ distribution studies

**Figure 6:**
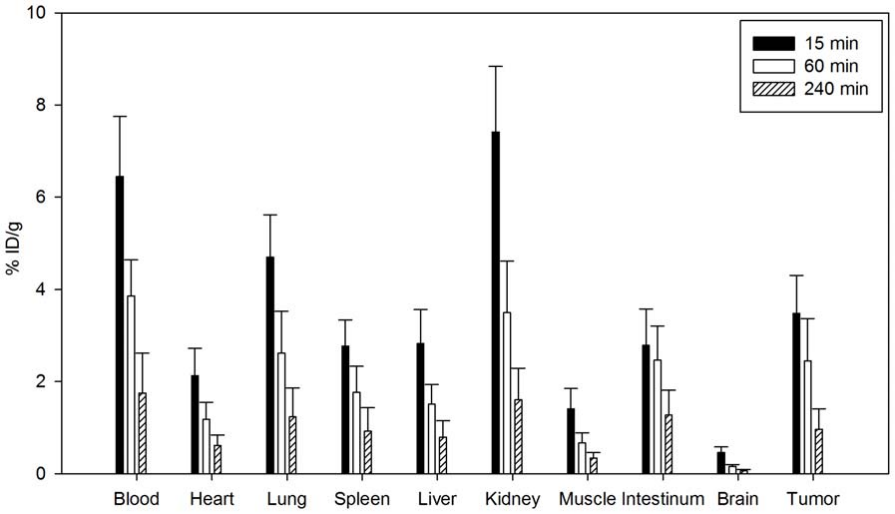
Organ distribution of ^131^I-labeled CaIX-P1 in female Balb/c nu/nu mice carrying SKRC 52 tumors. Activity concentration (% ID/g) in tumor and control organs is measured after 15 min (n = 8 animals), 60 min (n = 10 animals) and 240 min (n = 3 animals) circulation of ^131^I-labeled CaIX-P1 in the mice. Mean values and standard deviation.

Biodistribution experiments of ^131^I-labeled CaIX-P1 were performed in female Balb/c nu/nu mice, carrying subcutaneously transplanted SKRC 52 tumors. The organ distribution revealed a tumor uptake of 3.5% ID/g tissue at 15 min after intravenous injection of the radioligand. Uptake in tumor was higher than in heart, spleen, liver, muscle, intestinum and brain. Uptake in blood (6.4%) and the kidney (7.4%) was higher. After 60 min circulation a trend to a decrease of the uptake was noticed. This decrease was stronger for the healthy organs than for the tumor, resulting in an increase of the tumor-to-organ ratios (Table 1). Thereafter, a further decrease was noticed in tumor and healthy tissues (Figure 6).

**Table 1:** Tumor-to-organ ratios.

Tumor to organ Ratio	15 min	60 min	240 min	15 min blocking	60 min blocking
Blood	0.541±0.064	0.652±0.244	0.565±0.119	0.419±0.046	0.497±0.078
Heart	1.664±0.191	2.506±1.980	1.551±0.282	1.289±0.232	1.423±0.330
Lung	0.744±0.096	1.232±1.245	0.818±0.207	0.587±0.079	0.674±0.178
Spleen	1.258±0.142	1.585±1.122	1.095±0.240	1.007±0.143	1.152±0.117
Liver	1.248±0.138	1.813±1.095	1.239±0.223	0.907±0.121	1.226±0.232
Kidney	0.474±0.088	0.797±0.510	0.611±0.144	0.333±0.079	0.501±0.078
Muscle	2.529±0.310	4.111±2.444	2.781±0.599	1.884±0.463	2.623±0.264
Intestinum	1.299±0.301	1.396±0.546	0.859±0.492	1.145±0.069	0.864±0.226
Brain	7.736±1.066	17.60±9.740	17.19±5.018	6.631±1.725	9.71±4.034

Tumor-to-organ ratios were calculated from the organ distribution of ^131^I-CaIX-P1 in female Balb/c nu/nu mice carrying SKRC 52 tumors after 15 min (n = 8 animals), 60 min (n = 10 animals) and 240 min (n = 3 animals) circulation of the radioligand in the blood stream. For *in vivo* blocking, 100 µL unlabeled peptide at a concentration of 10^−3^ M was co-injected with the radioligand and tumor-to-organ ratios were calculated after 15 min (n = 3 animals) and 60 min (n = 3 animals) circulation in the blood stream.

*In vivo* blocking experiments with co-injection of unlabeled peptide demonstrated an uptake decrease in the tumor, but not in the healthy tissues, resulting in a decrease of the tumor-to-organ ratios. In particular, after 15 min circulation a decrease of about 25% for the tumor-to-muscle ratio was noticed (p<0.05) when the unlabeled peptide was co-injected, compared to the ratio without blocking. 60 min after intravenous injection, the tumor-to-muscle ratio was about 40% lower when the unlabeled peptide was co-injected, compared to the ratio without blocking. Tumor-to-organ ratios for all healthy tissues after blocking are presented in Table 1.

HPLC analysis of blood samples taken at 1 h post injection revealed that the majority of the radioactivity in blood was associated to serum proteins, while further amounts of free iodide and small peptide fragments were detected (data not shown).

## Discussion

Tumor hypoxia is known to be one of the key factors for malignant tumor aggression and progression, representing an independent negative prognostic factor for therapy outcome. Various experimental and clinical studies have confirmed the major role of hypoxia in treatment failure of both radiation therapy and chemotherapy [23] with an up to 3-fold resistance to radiation therapy. Oxygen deficiency leads to a reduced production of cytotoxic reactive species and promotes via accumulation of HIF-1α the upregulation of a variety of genes, such as glycolysis-associated genes or the vascular endothelial growth factor (VEGF), which not only induces angiogenesis but also protects the endothelial cells from irradiation [24].

The leading role of tumor hypoxia for therapy outcome and disease prognosis reveals the necessity for the development of hypoxia targeting and imaging assays. In the past years several tracers have been developed for hypoxia imaging using positron emission tomography (PET) [25]. Among them fluorine-18-labeled fluoromisonidazole (^18^F-FMISO) has been extensively evaluated in both preclinical and clinical trials demonstrating a significantly higher retention of the tracer in hypoxic than in normoxic tumors and a correlation between uptake and treatment response [26], [27].

A prominent intrinsic marker of tumor hypoxia is carbonic anhydrase IX (CAIX). CAIX is a tumor-associated member of the family of carbonic anhydrases that contributes to the acidification of extracellular pH and neutralization of intracellular pH protecting tumor cells from acidic pericellular microenvironment [28]. In this study we present the results of the evaluation of CaIX-P1, a new linear dodecapeptide with specificity for the extracellular domain of human carbonic anhydrase IX, identified through the technology of phage display. Binding experiments on the immobilized extracellular domain of CAIX revealed a higher accumulation on carbonic anhydrase IX, compared to the extracellular domain of the epidermal growth factor receptor, which was used as negative control target-protein. Tracer accumulation was found to be inhibited by the unlabeled CaIX-P1 peptide, which is evidence for a specific binding to the target. The hypothesis of a specific binding is supported by the results of the *in vitro* experiments, which demonstrated a higher accumulation in the known CAIX-positive SKRC 52 cell line [29], while the binding of the peptide was reduced to the background level for the cell line CaKi 2 which has been described as CAIX negative [29]. In addition, a dependence of binding of the radiolabeled peptide on the CAIX mRNA expression was shown for the colorectal carcinoma cell line HCT 116 and for human umbilical vein endothelial cells. Both mRNA expression of carbonic anhydrase IX and binding capacity of the CaIX-P1 peptide were higher for HCT 116 compared to the HUVEC cells. For the colorectal carcinoma HCT 116 cells the qRT-PCR analysis revealed a cell density dependent expression of CAIX, which also correlated to the binding of ^125^I-labeled CaIX-P1. Further evidence for a specific accumulation of the CaIX-P1 peptide was found in competition experiments revealing that the uptake of the radiolabeled ligand in CAIX positive SKRC 52 cells was reduced with increasing concentration of the unlabeled peptide, whereas octreotide as unspecific competitor at the same concentration had no effect.

The results of the internalization experiments demonstrated a quick internalization of the peptide at 37°C, which thereafter decreased with progression of time. Experiments at 4°C revealed a strongly reduced internalization to the background level. Those results are in concert with the results of previous studies, investigating CAIX specific antibodies. In particular, Chrastina et al. demonstrated a quick internalization of ^125^I-labeled monoclonal antibody M75, which is specific for human carbonic anhydrase IX, in human colorectal carcinoma cells [16]. Furthermore, the authors of this study point to the fact that antibodies, iodinated through the tyrosine residues, are dehalogenated after internalization and that the radioactive metabolites are excreted from the cells [16]. Such a process of intracellular dehalogenation or degradation, leading to radioactive products that are excreted by the cells, might also explain the time dependent reduction of both internalized activity and total cellular uptake of ^125^I-labeled CaIX-P1 at 37°C. The fact that the peptide CaIX-P1-2-12 shows different kinetics with slower decrease of the *in vitro* binding activity, allows the hypothesis that the radiolabeled N-terminal tyrosine residue is mostly affected by processes of *in vitro* dehalogenation or degradation, hypothesis which however has to be further investigated.

A prerequisite for the use of a ligand as tracer for imaging purposes is a higher *in vivo* accumulation in tumor tissue, compared to the healthy organs. Although organ distribution studies in nude mice bearing SKRC 52 tumors revealed a higher uptake in the tumor than in most of the healthy organs the blood values were higher, resulting in an enhanced background, which is a drawback for the use of the native peptide as imaging agent. With progression of time a reduction of the absolute uptake values in healthy organs and the tumor is noticed. This reduction is stronger in the healthy tissues than in the tumor for a circulation period of up to 60 min, resulting in an increase of the tumor-to-organ ratios. Thereafter however, a further uptake reduction in tumor and organs leads to a decrease of the tumor-to-organ ratios, which is disadvantageous for *in vivo* applications. The fast washout from the tumor is in concert with the results of the *in vitro* kinetic and internalization experiments and might also be explained by an intracellular dehalogenation or degradation process. Co-injection of unlabeled peptide in *in vivo* blocking experiments led to a significant reduction of the tumor-to-muscle ratios of about 25% after 15 min, which reached the level of about 40% after 60 min. Similar results were also revealed for other healthy organs, indicating an *in vivo* specificity of CaIX-P1.

The enhanced blood values are explained through an interaction of the peptide with serum proteins, such as albumin or through *in vivo* deiodination of the radioligand. HPLC analysis of blood at 1 h after injection of ^131^I-labeled CaIX-P1 revealed that the majority of the radioactivity was associated to serum proteins. In addition, free iodide and small peptide fragments were detected. *In vivo* deiodination of directly radiolabeled peptides has been described in the literature [30]. This problem can be adressed through chemical modifications of the peptide. A possible way to enhance resistance to deiodination of peptides is the protection of the radioiodinated N-terminal tyrosine with a t-butyloxycarbonyl group [31]. Alternatively, further radiolabeling approaches, such as metal labeling through a chelator might be applied in order to improve labeling stability and reduce radioactive background [32]. In case of CaIX-P1 the high blood value is additionally explained by the metabolic properties of the peptide. Stability experiments in human serum demonstrated a degradation of CaIX-P1 through serum proteases. Mass spectrometry revealed a degradation of a tyrosine molecule. Since direct iodination is performed on the side group of tyrosine, the degradation might lead to free ^125^I-labeled tyrosine residues that circulate in the bloodstream. In this way the organ distribution of CaIX-P1 is negatively influenced by both radiolabeling and metabolic instability. Therefore, a major issue of further investigation is the serum stabilization of the CaIX-P1 peptide. Methods to achieve improved serum stability are synthesis of shorter derivatives of the peptide, exchange of amino acids by unnatural amino acids that can not be recognized by serum proteases, acetylation or pegylation of the molecule [33], [34] and grafting of the binding motif into a stable scaffold structure [35]. Such modifications might improve the metabolic properties of the peptide, increase its affinity and binding capacity on the target structure and lead to enhanced tumor-to-organ ratios, which is of high importance for the development of *in vivo* targeting and imaging strategies.

In conclusion, peptides with affinity for the tumor associated carbonic anhydrase IX can be used as lead structures for targeting of imaging agents in hypoxic tumor sites. The evaluation of the newly identified peptide CaIX-P1 indicates that the peptide might be a promising candidate, which could be used as lead structure for the development of new tracers with affinity for human carbonic anhydrase IX. Based on the results of the *in vitro* experiments the hypothesis of a specific binding to the target can be generated. However, the organ distribution studies demonstrate low tumor-to-blood ratios, which is disadvantageous for the clinical use of the native ligand for imaging purposes. Therefore, further studies are needed in order to improve the serum stability of CaIX-P1, optimize its binding efficacy and lead to generation of peptide-based ligands, which can be used for targeting human carbonic anhydrase IX and tumor hypoxia.

## Supporting Information

Figure S1 ***In vitro*** **kinetics of ^125^I‐labeled‐CaIX‐P1‐2‐12.** Incubation of the radiolabeled first metabolic product of CaIX‐P1 (CaIX‐P1‐2‐12) was performed on CAIX positive SKRC 52 cells for time periods from 10 min to 120 min. Mean values and standard deviation (n=3).
(TIF)
