# Supplementary figures and images for: A New Peptide Ligand for Targeting Human Carbonic Anhydrase IX, Identified through the Phage Display Technology
Source: PLoS One. 2010 Dec 31;5(12):e15962. doi: 10.1371/journal.pone.0015962 (PMC3013143; doi:10.1371/journal.pone.0015962)

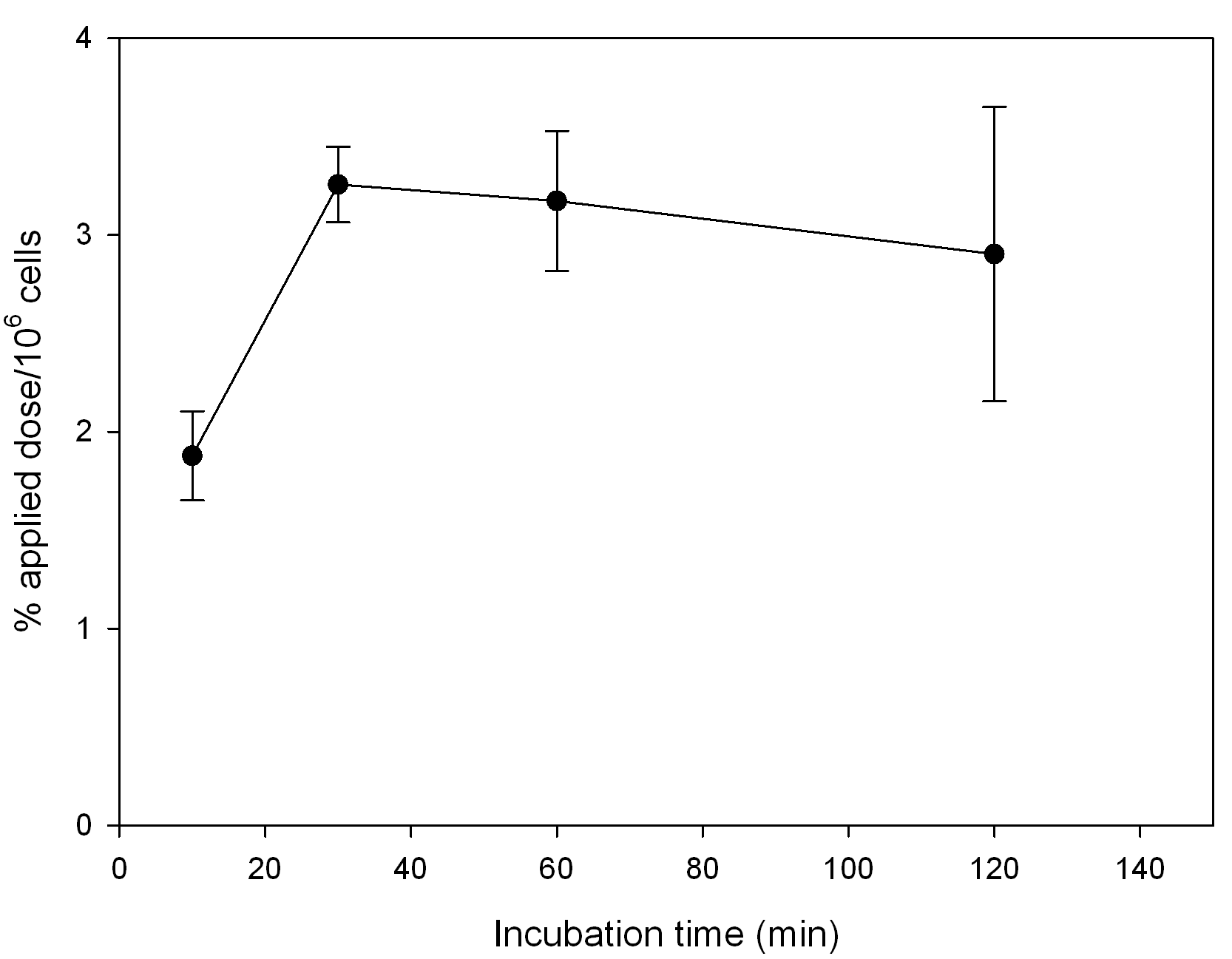

Supplement: Figure S1 — In vitro kinetics of 125I‐labeled‐CaIX‐P1‐2‐12. Incubation of the radiolabeled first metabolic product of CaIX‐P1 (CaIX‐P1‐2‐12) was performed on CAIX positive SKRC 52 cells for time periods from 10 min to 120 min. Mean values and standard deviation (n=3). (TIF) [file pone.0015962.s001.tif]
